# Combined Experimental and Numerical Modelling of the Electrical Behaviour of Laser-Soldered Steel Sheets

**DOI:** 10.3390/ma17112736

**Published:** 2024-06-04

**Authors:** Andor Körmöczi, Gábor Horváth, Tamás Szörényi, Zsolt Geretovszky

**Affiliations:** Department of Optics and Quantum Electronics, University of Szeged, 6720 Szeged, Hungary; kandor@titan.physx.u-szeged.hu (A.K.); gero@physx.u-szeged.hu (Z.G.)

**Keywords:** laser soldering, filler material, lap geometry, electrical measurement, numerical modelling

## Abstract

The electric vehicle (EV) industry challenges battery joining technologies by requiring higher energy density both by mass and volume. Improving the energy density via new battery chemistry would be the holy grail but is seriously hindered and progresses slowly. In the meantime, alternative ways, such as implementing more efficient cell packaging by minimising the electrical resistance of joints, are of primary focus. In this paper, we discuss the challenges associated with the electrical characterisation of laser-soldered joints in general, and the minimisation of their resistive losses, in particular. In order to assess the impact of joint resistance on the overall resistance of the sample, the alteration in resistance was monitored as a function of voltage probe distance and modelled by finite element simulation. The experimental measurements showed two different regimes: one far from the joint area and another in its vicinity and within the joint cross-section. The presented results confirm the importance of the thickness of the filler material, the effective and total soldered area, and the area and position of the voids within the total soldered area in determining the electrical resistance of joints.

## 1. Introduction

Electric vehicles (EVs) have gained significant attention as green and environmentally friendly alternatives to vehicles driven by internal combustion engines [1,2,3,4]. A key component driving the propulsion of EVs is the battery pack, which stores and supplies electrical energy to power the vehicle’s electric motor. Battery packs play a critical role in determining the driving range, performance and overall environmental impact of both fully electric and hybrid vehicles [5,6,7,8]. As the demand for EVs continues to rise, there is a pressing need to increase the energy storage capacity of the battery pack, as well as to optimise its design, efficiency, and packaging [9,10].

While novel battery chemistry presents a promising, long-lasting avenue to meet the needs of vehicle manufacturers, its transformative impact is hindered by the intrinsic limitations of time-intensive development [11,12]. Considering this, a complex strategy is outlined that involves all aspects that maximise the extractable energy from the cells/battery packs making use of current battery chemistry [13]. Central to this strategy is the smart management of losses encountered during cell interconnections [14,15]. The reduction of such losses bears significant potential and may/can substantially contribute to overall efficiency. The extent of energy loss is primarily determined by the resistance of individual joints, which makes it essential to both accurately characterise and minimise these resistive losses. Therefore, the development of two things is important: a sturdy, well-controlled and reproducible joining process and an appropriate resistance measurement methodology for, at least, comparative purposes.

In recent years, from the plethora of potential joining technologies, laser-based welding, soldering, and brazing have emerged as particularly promising alternatives to traditional bonding methods, offering the potential for producing high-quality joints. Laser-assisted filler-based joining techniques, such as soldering and brazing, have even surpassed laser welding in terms of electrical properties [16,17]. Brand et al. showed that soldered joints exhibited the lowest resistance compared to other joining technologies, such as resistance, ultrasonic, and laser welding [17].

On the other hand, the electrical characterisation of joints in overlap geometry created by any technique is a challenging task in itself, and no standardised methods are currently available to meet these. On most occasions, the four-point probe method, also known as the Kelvin or van der Pauw method is the most favourable option [16,17,18,19,20,21], as it offers a robust approach for resistance measurement that is also capable of mitigating measurement errors introduced by contact and lead wire resistances. The four-point probe method enables precise determination of the joint’s resistance while minimising the impact of parasitic resistances, resulting in highly accurate and reproducible measurements [18]. However, the measurement probes are unable to distinguish between the resistance of the joint and the resistance of the base sheets to be joined, regardless of how close they are to the joint interface. In the literature, the four-point probe method with several distances between the needle-tipped probes was used. Schmidt et al. and Schmalen et al. used 21 mm as a distance between the probes [16,17,20], while a 16.5 mm or 11 mm distance was used by Biele et al. [18] and Hollatz et al. [21], respectively. The method, performed at one fixed measurement distance, is perfectly suitable for comparing the electrical characteristics of different samples but it cannot give exact values for the joint; instead, it provides the cumulative resistance of the base plates and the filler together.

We could not find any report describing an in-depth analysis of the electrical characteristics of laser-soldered connections. Drawing upon all the information currently available in the literature, we suggest using laser soldering as a joining technology for battery connection, and our approach is to find an evaluation strategy that is capable of providing the critical parameters of the electrical behaviour of these joints. Therefore, in this paper, we report on electrical measurements of laser-soldered DC01 and Hilumin^®^ sheets by monitoring the resistance as a function of the spatial distance between the two voltage probes. In addition to the experimental results, this study incorporates results obtained in the COMSOL Multiphysics [22] environment to enhance the understanding of the electrical behaviour of connected sheets.

## 2. Materials and Methods

Joints were established between two types of steel sheets, specifically DC01 steel sheets measuring 0.5 mm in thickness, 15 mm in width, and 30 mm in length, and Hilumin^®^ sheets with a thickness of 0.15 mm, a width of 9 mm, and length of 30 mm. Hilumin^®^ is a protected trademark of Tata Steel (London, UK) for sheets specifically developed for battery applications and is a widely preferred material of the caps of cylindrical cells. It is made by electroplating a cold-rolled battery quality steel (DC04) with a nickel layer of several micrometres’ thickness with a final diffusion annealing step, which results in low contact resistance and high corrosion resistance [23].

**Figure 1:**
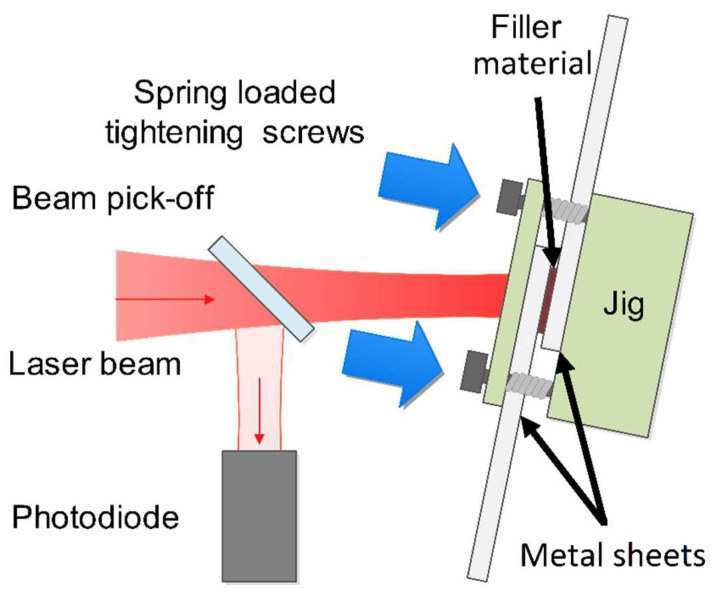
Schematic illustration of laser soldering of two metal sheets.

The joint configuration chosen for our study was overlap geometry, since it represents well the ”cap to cap,” “bus bar to bus bar,” and “battery cap to bus bar” joint types and is hence responsible for the majority of the joints commonly employed in the assembly of a battery pack. To facilitate the connection of the two steel sheets, a homemade jig was used to clamp the plates together. While the possibility of using different Sn-Cu solders was considered, Sn99.3Cu0.7 was chosen. The tin-based filler was positioned between the two steel pieces, as illustrated in Figure 1. The filler material was also used in the form of sheets, with a thickness of 0.16 ± 0.01 mm and a width of 5.0 ± 0.1 mm. To investigate the effect of filler amount, the length of the filler sheet was varied between 2.0 and 10.0 mm in the case of DC01 samples, and between 0.5 and 4.0 mm in the case of Hilumin^®^ samples. It is important to note that the chosen tin-based filler had a liquidus temperature of 227 °C and incorporated a rosin-based flux to ensure proper wetting of the soldered surfaces [24]. Before fixing the sheets into the jig, both steel sheets were roughened with p1200 sandpaper and precleaned with acetone rinsing.

A continuous-wave Yb-doped fibre laser (Model SP-400C-0005, SPI Lasers plc, Southampton, UK (currently Trumpf)) was utilised as the heat source. The laser emits a maximum power of 400 W at the central wavelength of 1071 nm and exhibits an almost perfectly Gaussian beam with an M^2^ value of approximately 1.08. The laser beam was directed onto the sample surface at an angle of incidence of approximately 10° in order to avoid the back reflection of the laser light into the cavity. For the soldering process, a glass lens with a focal length of 1000 mm was utilised to produce an illuminated spot in the convergent laser beam phase on the upper surface of the sheet pair, creating a slightly elliptical irradiation spot with a minor axis of ~4.5 mm, defined by the 1/e^2^ intensity criterion. To measure the laser power in situ, a photodiode (Thorlabs, Newton, NJ, USA, S130C) sampled a small portion of the processing laser beam to facilitate accurate and real-time power measurements during the experiments.

**Figure 2:**
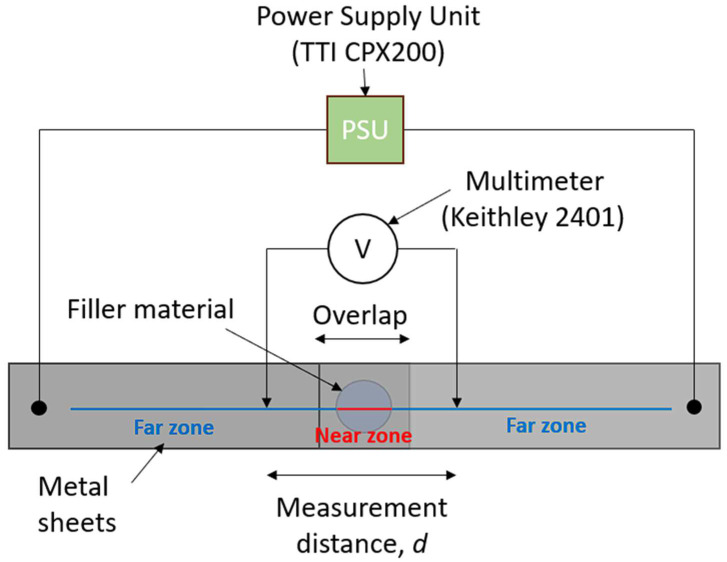
Schematic illustration of the electrical measurement setup. Far and near zones are marked.

Measuring the sole resistance directly of the joint is a challenge because the resistance of the two sheets to be joined will always contribute to the measured net resistance, no matter how closely we approach the actual contact area with the measuring probes. For measuring accurately the electrical resistance of the welds, the four-point probe measurement geometry was used, minimising errors introduced by parasitic resistances. The current was fed by a TTi (Austin, TX, USA) CPX200 power supply at the ends of the plates to be joined at a distance of 100 mm, and the voltage drop was measured across two internal probes by a Keithley (Cleveland, OH, USA) 2401 multimeter so that the geometric centre of the joint coincided with the middle point of the voltage measurement distance, as depicted in Figure 2. This method is perfectly suitable for comparing the electrical characteristics of different samples if the distance of the internal electrodes is kept constant. However, it does not give exact values for the joint; instead, it provides a cumulative resistance. Therefore, the measurement distance was varied between 1 and 81 mm in this study.

One way to accurately determine small resistances is to use large test currents in order to produce a voltage drop that can be measured with sufficient accuracy. As an example, to determine a 15 µΩ resistance with a 1% measurement error, a current of at least 180 A is required [25]. However, such high currents are likely to cause a temperature rise within the samples that will affect the resistance, and ultimately may even cause damage in the structure of the joint. Therefore, we needed an alternative solution to precisely measure the resistance of the laser-produced joints. One possible solution, coined the *asymptotic method*, is where the resistance of the joint is measured at several current values and the resistance is plotted as a function of current. Here, the resistance is the limiting resistance value to which this *R(I)* curve approaches. Another approach, the *slope method*, is more accurate. Here, the potential drops are measured at several currents, and the slope of the *U(I)* curve provides the resistance of the joint. An additional advantage of the latter method is that it is “self-checking”: if all points fit well into a straight line, it proves that during the measurement, none of the applied currents causes a temperature rise in the system (i.e., the resistance is constant). Therefore, all resistance values reported here were measured by the *slope method* after carefully testing the linearity of the individual *U(I)* datasets. The maximum current value that allowed measurements without any noticeable temperature rise during the 1–2 s measuring time was set as 10 A. We found that, for keeping the error of the resistance values below 1%, measuring at 6 measurement points was necessary for the 0.1–10.0 A current range. Therefore, we derived joint resistances by measuring the potential drops at 0.1, 0.5, 1.0, 3.0, 6.0, and 10.0 A currents.

Our experimental investigations were complemented with numerical modelling. By developing a model in COMSOL Multiphysics, we gained insight into the distribution of current density and electric potential (by which the resistance can be calculated) within and in the vicinity of the soldered joint, facilitating a deeper understanding of the electrical characteristics of the interconnected sheets. Here, the challenge is how well the COMSOL model describes the real joint in terms of its structure (e.g., electric transfer at the interfaces) and the material property of the relevant component (sintered metal sheets or resolidified solder). The computational framework COMSOL Multiphysics version 5.5 was used for integrating three key modules, including the CAD Import Module, Library Material, and AC/DC Module. The accurate representation of the material behaviour was realised through the measurement and subsequent integration of pertinent electrical properties into the model. Notably, the foundational material for these properties was established based on the predefined “iron” and “Sn—1 Cu” tin-based filler material from the Library Material module, thereby ensuring a robust and consistent starting point. The modelling of the electrical contact of the surface between the materials is a complex problem. To solve this, we employed one of the modules available in COMSOL, which is based on the Cooper–Mikic–Yovanovich (CMY) correlation [26]. The CMY correlation takes into account the roughness and microhardness of the surface and pressure load at the contact interface. Therefore, we measured the microhardness and roughness of the surface throughout our study as well. The Vickers microhardness was found to be (244.16 ± 2.47) HV0.1, and the average surface roughness, according to the ISO 4287:199 standard [27], was (2.20 ± 0.13) µm. To achieve a high level of accuracy and resolution in our simulations, the predefined “extremely fine” mesh was employed throughout the computational calculations. In COMSOL Multiphysics, the iterative numerical processes within the solver will continue to iterate on the solution until the calculated relative error approximation drops below the prespecified relative tolerance. The relative tolerance is computed as the weighted Euclidean norm of the resistivity vectors [28]. For all the cases reported in the present study this relative tolerance was set to 0.001.

## 3. Results and Discussion

To assess the impact of joint resistance on the net resistance of the sample, a thorough investigation was conducted. Instead of deriving the resistance at one fixed probe distance, the resistance, *R,* was monitored as a function of the spatial distance between the two voltage probes (to be denoted as voltage probe distance, *d*).

**Figure 3:**
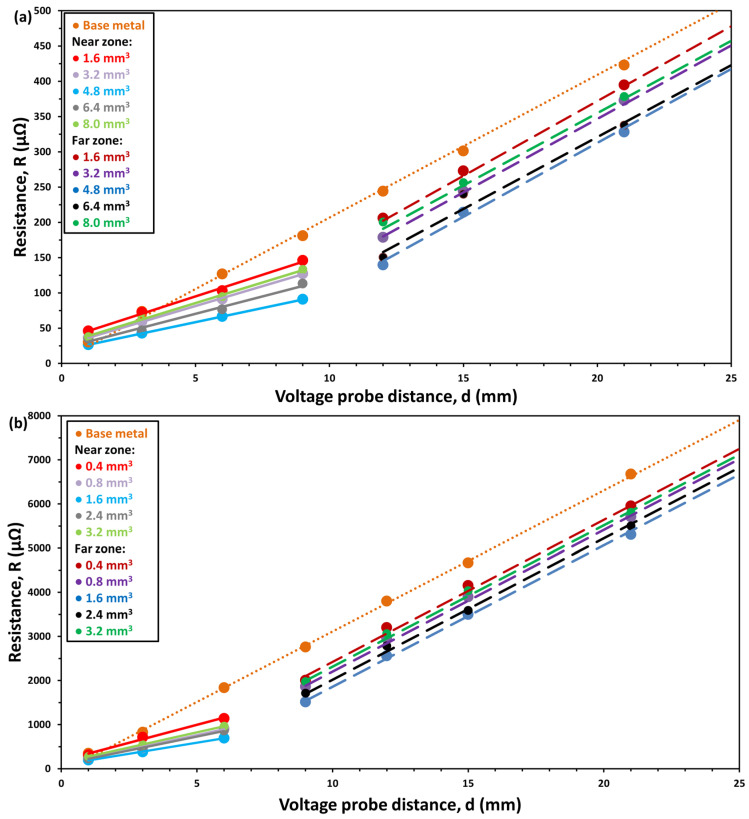
Resistance as a function of voltage probe distance for different amounts of filler materials for (**a**) DC01 and (**b**) Hilumin^®^ samples.

The analysis of these *R(d)* curves exhibited two zones (cf. Figure 2) according to the different dependence of the resistance on the voltage probe distance. The boundary line between these two zones correlated well with the edge of the joining area (i.e., the perimeter within which resolidified filler was present after separating the joint sheets). Figure 2 proves the spatial distinction between the far and near zones. In the far zone (indicated by the blue lines in Figure 2), the measurement probes were positioned outside of the joining area. On the other hand, the near zone (marked by the red line in Figure 2) encompassed measurements conducted within the joining area. In Figure 3, the near and far zones are distinguished by full and dashed lines.

The net resistance as a function of the voltage probe distance at several different filler material volumes is shown in Figure 3a,b for the DC01 and Hilumin^®^ samples, respectively. The DC01 samples were processed with 120 W laser power at 5 s irradiation time, while the Hilumin^®^ samples were joined with 80 W laser power at 5 s.

**Figure 4:**
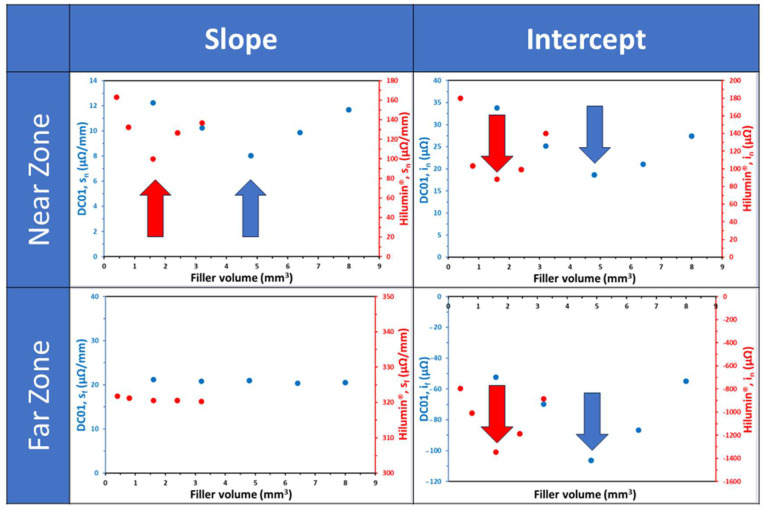
Fitting parameters in far and near zones for DC01 (blue) and Hilumin^®^ (red) sheets as a function of the volume of the filler material. The optimal filler volume is indicated by the arrows.

Both material series exhibit comparable trends; therefore, we elaborate upon the properties primarily within the context of the DC01 series. Across all soldered samples in the series, a consistent pattern emerges, where the resulting resistance is consistently lower than that of the base material. A distinction in the *R(d)* relationship is apparent between the near and far zones. Moreover, a nearly linear correlation between the resistance and the voltage probe distance is discerned within both individual zones. Furthermore, this linearity persists regardless of the quantity of filler material used. It is also clear that every *R(d)* curve breaks between 9 and 12 mm and 6 and 9 mm for DC01 and Hilumin^®^, respectively. These breakpoints correlate with the diameter of the effective soldered area. Nevertheless, the slope and the intercept of these linear functions are dependent upon the volume of the filler material, as shown in Figure 4. Notably, the dependencies from the volume do not follow a monotonous trend; rather, these curves moved further and further away from the straight line of the base metal (i.e., they had a smaller intercept by increasing the amount of solder). This trend could be seen until the optimum amount of solder (indicated by arrows in the figure) was reached, after which it reversed. This behaviour is fully in line with the morphological variation of the resolidified filler reported earlier [29], namely that there is an optimal amount of solder for any laser power–irradiation time pair, where the filler completely melts and results in a joint that exhibits the most homogenous resolidified filler (i.e., the one which has the least amount of voids (due to bubbles) and/or solid remnants). This optimum is 4.8 mm^3^ and 1.6 mm^3^ for the DC01 and Hilumin^®^ sets, respectively, under the process conditions applied. While the trends are congruent, differentiation arises in the parameter values that define the linear relations encapsulating the electrical behaviour between the DC01 and Hilumin^®^ series. The variations in the slope and the intercept inherently distinguish the DC01 and Hilumin^®^ material series, thereby supporting the fact that they have unique electrical properties.

## 4. Modelling and Discussion

Numerical simulations provide a valuable tool for studying complex phenomena and understanding the underlying physics of systems in a controlled virtual environment where experimental manipulation is challenging. By comprehensively exploring the effect of factors such as the resistivity of the materials to be joined and filler material geometry, a three-dimensional finite element model was developed using the COMSOL Multiphysics^®^ 5.5 program.

**Figure 5:**
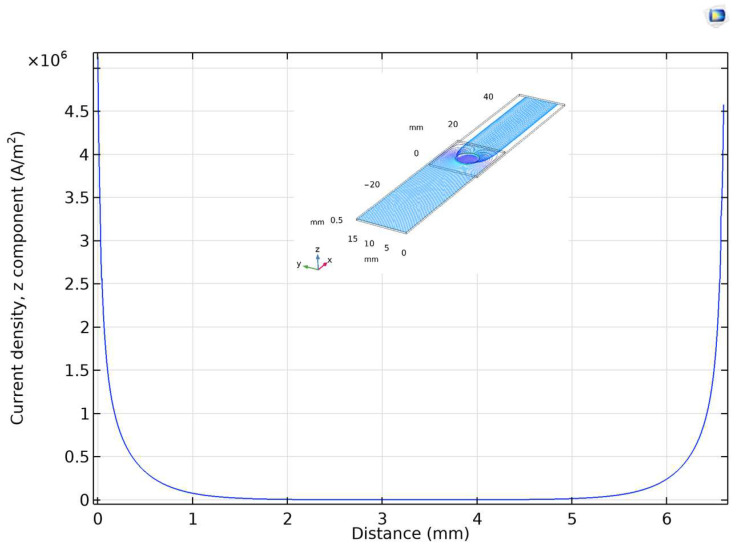
The *z* component of the current density vectors in filler material at the *y* = 7.5 mm and *z* = 0.53 mm sections, as shown in the inset.

**Figure 6:**
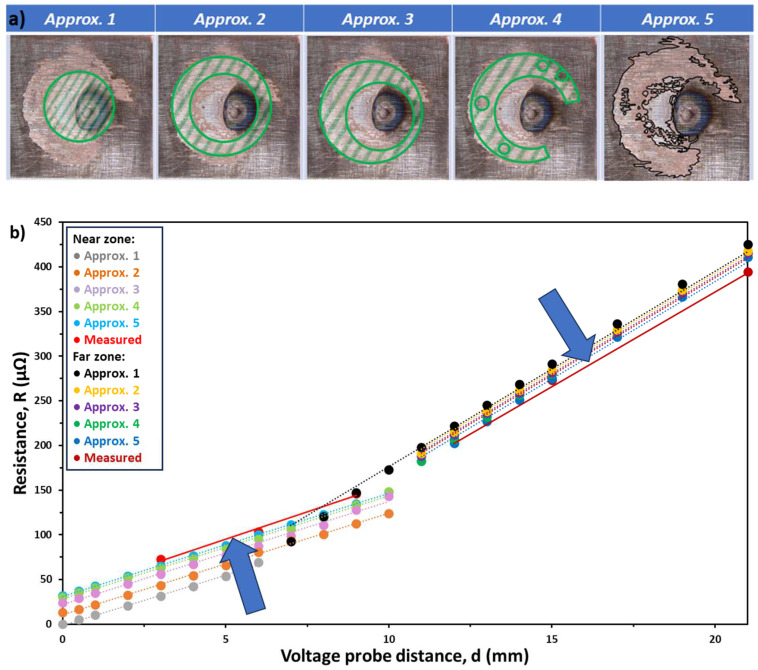
(**a**) Five different approximations of the real geometry of the resolidified filler material, and (**b**) the result of these approximations compared to a measured dataset (solid lines).

To check the validity of the model, the distribution of the current density vectors over the contact area was determined first. The three-dimensional COMSOL model revealed that approx. 97% of the current flowed through the 10% outermost part of the full contact area (Figure 5). This result is in line with the conclusion of a study by Brand et al., who described soldered joints with a simplified equivalent circuit model (called a “resistance network”) with the characteristics given analytically in two dimensions [17]. As the next step, the effect of a filler of 1.6 mm^3^ volume on the electrical resistance of the joint was simulated for four different forms of the resolidified filler material while the effective bonding area was kept constant as follows: cylindrical filler (*Approx. 1.*), cylindrical filler with one big void (*Approx. 2.*), cylindrical filler with a big void in an asymmetric position (*Approx. 3.*), and cylindrical filler with a big asymmetrical void and several smaller voids (*Approx. 4.*). Finally, the precise geometry of a real joint was modelled in 3D using the optical microscope image of the torn surface (*Approx. 5.*). These five models are depicted in Figure 6a and the results of the simulated *R(d)* curves are shown in Figure 6b.

These numerical simulations predict a linear dependence of the resistance on the voltage probe distance (fully in line with the experimental behaviour presented in Figure 3) in both near and far zones. It is also clear that every *R(d)* curve breaks at around 11 mm (except at *Approx. 1.*, where the diameter was 6.6 mm); specifically, the precise diameter of the inbounding circle used to describe the filler in the 3D model. However, there is a deviation between the measured and simulated *R(d)* curves: the calculated *R* values are continuously smaller and larger than the measured ones in the near and far zones, respectively. Moreover, the refinement of the geometrical fidelity of the resolidified filler (through *Approx. 1. to 5.*) results in simulated *R* values that approach the measured ones (plotted in solid lines) from below and above, in the near and far zones, respectively (indicated by the blue arrows). Near and far linearities do not depend on the geometry of the filler, while the role of the geometry is decisive in the accuracy of the specific resistance values. Furthermore, the approximations resulted in bigger differences in resistance in the near zone compared to the far zone.

Experimenting with these numerical simulations and especially with the different approximation models proved that five geometrical properties of the filler material influenced the electrical characteristics, namely *the thickness of the resolidified filler material* between the two sheets, *h*; the area where the two metal sheets are joined by the filler, referred to as *effective soldered area, A_eff_*; the *total area of voids, A_void_*; the *total joint area, A_total_*, which is the sum of the effective soldered area and the area of the voids; and finally, the *position of the voids within the total soldered area*. In the following, the effect of these five parameters on the linear functions in the near and far zones is shown for the case of connecting the two DC01 stripes with a filler of 1.6 mm^3^ volume. The reason for choosing this case was that, among the samples of different volumes of filler, this one contained the most voids, hence being the most appropriate to model the effect of the void area and the effect of their distribution. Five cases were considered:

- *Case I*: The thickness of the resolidified filler material was varied, while we kept the filler volume constant (*t_filler_* = *varied*, *V_filler_* = *constant*).
- *Case II*: The effective soldered area was varied without the presence of voids (*A_eff_* = *varied*, *A_void_* = 0).
- *Case III*: The total joint area was kept constant, while its two components (i.e., the effective soldered area and the void area) were varied (*A_total_* = *A_eff_* + *A_void_* = *constant*, *A_void_/A_total_* = *varied*).
- *Case IV*: The effective soldered area was fixed while varying the void area, which resulted in the variation of the total soldered area (*A_void_* = *varied*, *A_total_* = *varied*, *A_eff_* = *constant*).
- *Case V*: The distribution of voids (i.e., their location was shifted within the total soldered area; *A_total_* = *constant*, *A_void_* = *constant*, *pos_void_* = *varied*).

## 5. The Effect of Characteristics of the Soldered Joint on the *R(d)* Function

In order to determine the effect of the soldered joints on the characteristics of the *R(d)* plots, the simulated parameters were changed from −50% to +50% relative to the measured value of each parameter (except *Case V*). Table 1 summarises the experimentally obtained values of each and every parameter of the soldered joint listed above, together with their respective ranges in which we varied their values in the simulations.

The relative changes of the minima to the maxima of the slopes were expressed in percents with the following equation:

$$\delta =\frac{X_{max}-X_{min}}{X_{max}}$$

where *X* stands for the slopes (*s_f_*, *s_n_*) or the intercepts (*i_f_*, *i_n_*) in the far and near zones. The negative or positive sign of the relative change indicates that when the simulated parameter increases (i.e., from −50% to +50% of the measured value), the calculated slopes move away from or closer to the measured characteristics, respectively. Varying the property resulted in monotone changes in each and every case. Two types of changes were distinguished: linear and reciprocal.

The distance dependence of the resistance, *R(d)*, is a linear function that will be described in the following form for both the near and far zones:

$$R_{x}\left(d\right)=s_{x}\cdot d+i_{x}$$

where *s_x_* and *i_x_* denote the slope and the intercept of the straight line, and the *x* index can be *f* for far and *n* for near zone.

### 5.1. The Far Zone

**Figure 7:**
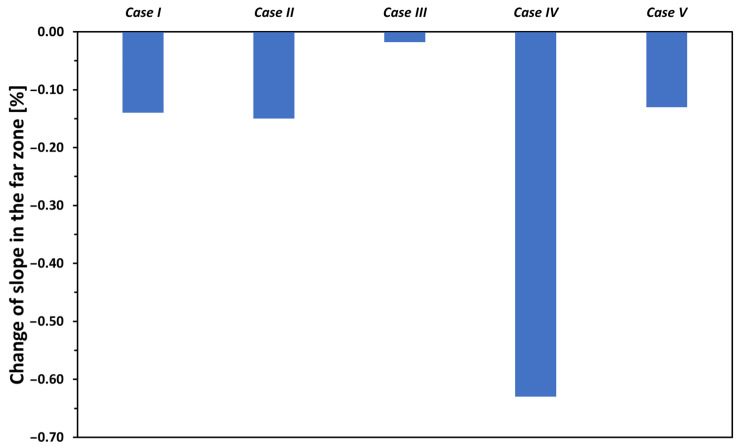
The effect of variation of the five relevant properties of the soldered joint by ±50% on the slopes in the far zone.

The relative changes of the slopes for different cases in the far zone are shown in Figure 7. The magnitude of the relative changes is small (i.e., less than 0.65% in all cases); none of the parameters varied have a substantial impact on the slope in the far zone.

Previously, we concluded that the measured slope in the far zone was the same for all amounts of filler materials but differed between the DC01 and Hilumin^®^ samples. This observation was consistent with the conclusion that the far-zone slope was determined by the material of the sheets to be joined. This point was double-checked by measuring the slope of the *R(d)* curves in the far zone with different metal sheets. It can be demonstrated algebraically that

$$s_{meas.}=\frac{R(d)}{d}=\frac{\rho_{sheet}}{A_{sheet}}$$

The experimentally determined resistivity values derived from the measured slopes and the cross-section of the sheets exhibit a close correlation to the values reported in the literature, as summarised in Table 2. Therefore, we conclude that the slope of the *R(d)* curves in the far zone is related to the resistivity of the material to be joined.

We double-checked the effect of the cross-section of these sheets on the far-zone slope of the *R(d)* functions, as well. When the width of the sheet was halved, the slope was increased by two times. Therefore, the slope in the far zone, *s_f_*, can be described by the following formula:

(1) $$s_{f}=\rho_{DC01}/A_{DC01}$$

where *ρ_DC01_* is the electrical resistivity of the sheet’s material and *A_DC01_* is the cross-section of the metal sheets to be joined. Therefore, the final conclusion is that the slope in the far zone is determined by the electrical resistivity and the cross-section of the material to be joined.

**Figure 8:**
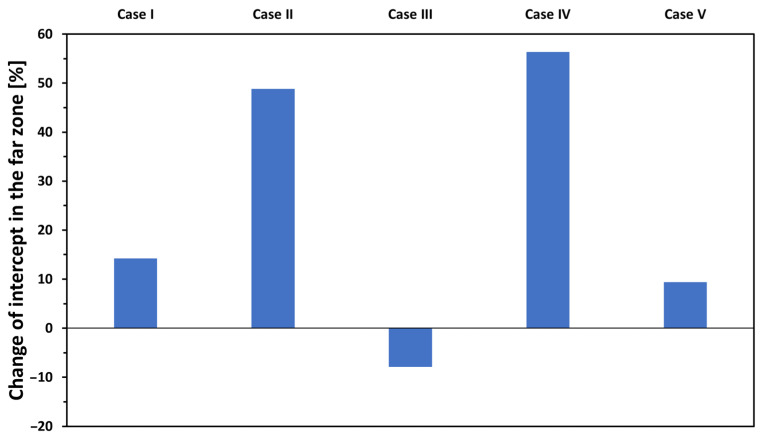
The effect of changing the five relevant properties of the soldered joints by ±50% on the intercept of *R(d)* in the far zone.

**Figure 9:**
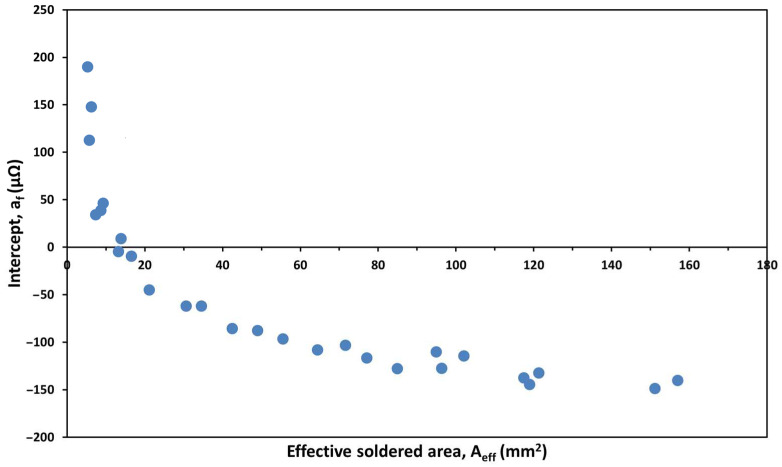
The intercept of the *R(d)* in the far zone, as a function of effective soldered area (the voltage probe distance was set to 21 mm in this case).

The results of simulations expressed as relative changes that refer to the behaviour of the intercepts in the far zone are shown in Figure 8. The intercepts are dominantly determined by the *effective soldered area (Case II)*, and the *total joint area (Case IV)*. These two parameters have a five-times larger effect on the intercept than any of the other three parameters. The calculated intercepts derived from the differences between the resistance of the sheet and the respective joints measured at 21 mm are displayed as a function of the effective soldered area in Figure 9. The intercepts decrease with increased effective soldered area and changes sign at approx. 15 mm^2^ soldered area. The rate is most significant in the 0–25 mm^2^ area domain and flattens off at and above 60 mm^2^, ultimately approaching an intercept value of approx. −150 µΩ. These results, in line with the model calculations, reveal a reciprocal dependency from the effective soldered area with an exponent of 0.65 up to flattening:

(2) $$s_{f}=\rho_{DC01}/A_{DC01}$$

An increase in the volume of the voids (*Case III*) is detrimental by increasing the resistance of the joint, as demonstrated experimentally [29], appearing as a negative relative change in the intercept. These two effects, dominated by effective soldered area (*Case II*), are combined in the impact of the total joint area (*Case IV*).

### 5.2. The Near Zone

**Figure 10:**
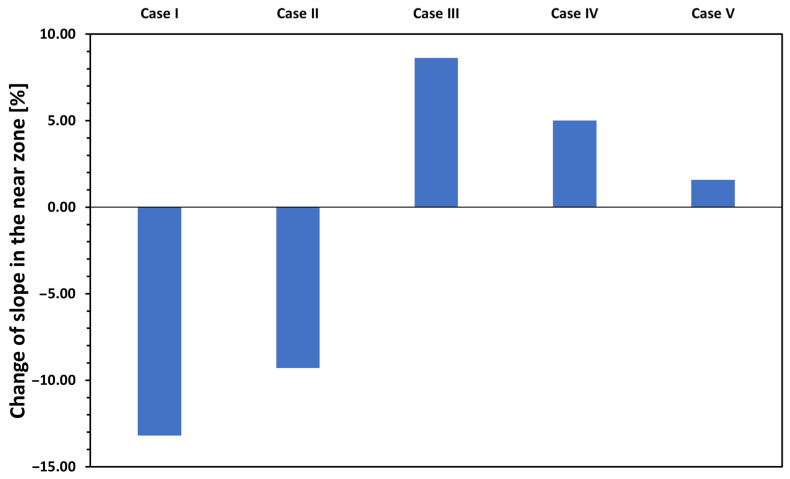
The effect of changing the five relevant properties of the soldered joint by ±50% on the slope of the *R(d)* function in near zone.

In order to determine the effect of the five relevant geometrical properties of the filler material on the slope in the near zone, the parameters were changed similarly to those in the far zone. The relative effects observed in the five cases are shown in Figure 10.

In the near zone, the change of each and every property of the soldered joint causes a change of at least one order of magnitude greater than in the far zone. Among the five parameters examined, the *void area (Case III)* emerges as the most influential parameter in positive and the *thickness (Case I)* and the *effective soldered area (Case II)* in a negative way (i.e., whether the modelled points are closer or further away from the measured slope data points). These changes mean that the addition of filler material and volume of voids results in overall lower and higher resistance in the near zone, respectively.

**Figure 11:**
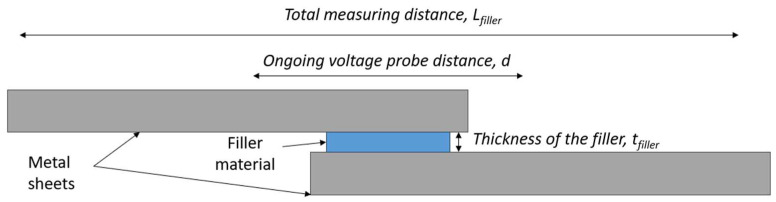
Sketch of the joint indicating the quantities defining the *c*_1_ and *c*_2_ coefficients.

If we assume that the two sheets and the filler are connected in parallel, then

(3) $$s_{n}\approx {\left(\frac{A_{DC01}}{c_{1}\cdot \rho_{DC01}}+\frac{A_{eff}}{c_{2}\cdot \rho_{filler}}\right)}^{-1}$$

where *ρ_filler_* is the electrical resistivity of the filler, and the *c*_1_ and *c*_2_ are coefficients that are related to the geometry of the sheets and the filler (cf. Figure 11):

$$c_{1}\approx \left(L_{filler}-t_{filler}\right)-\left(L_{filler}-d\right)=d-t_{filler}\;\mathrm{and}\;c_{2}\approx t_{filler}$$

While being formally similar to Equation (1), valid in the far zone, this definition of the slope by Equation (3) is more universal, since it combines the contribution of both the metal sheets and the filler material. In the far zone, the voltage probe distance, *d*, is much greater than the thickness of the filler; hence, *c*_1_ >> *c*_2_, the second term in Equation (3) describing the slope in the near zone, can be neglected. Therefore, Equation (3) can be taken as a universal definition of the slope that applies both in the near and far zones.

**Figure 12:**
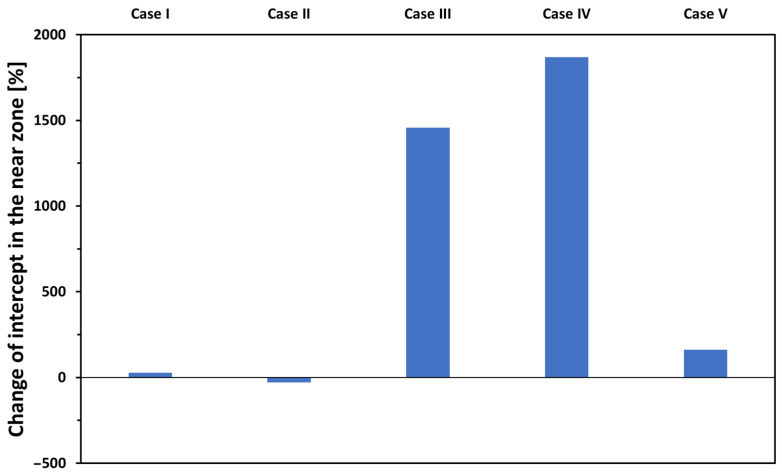
The effect on the intercept in the near zone of the five relevant properties of the soldered joint when changing them ±50% in relative terms to the measured data.

The results displaying the impact of the five relevant properties of the filler material’s geometry on the intercept in the near zone are shown in Figure 12.

**Figure 13:**
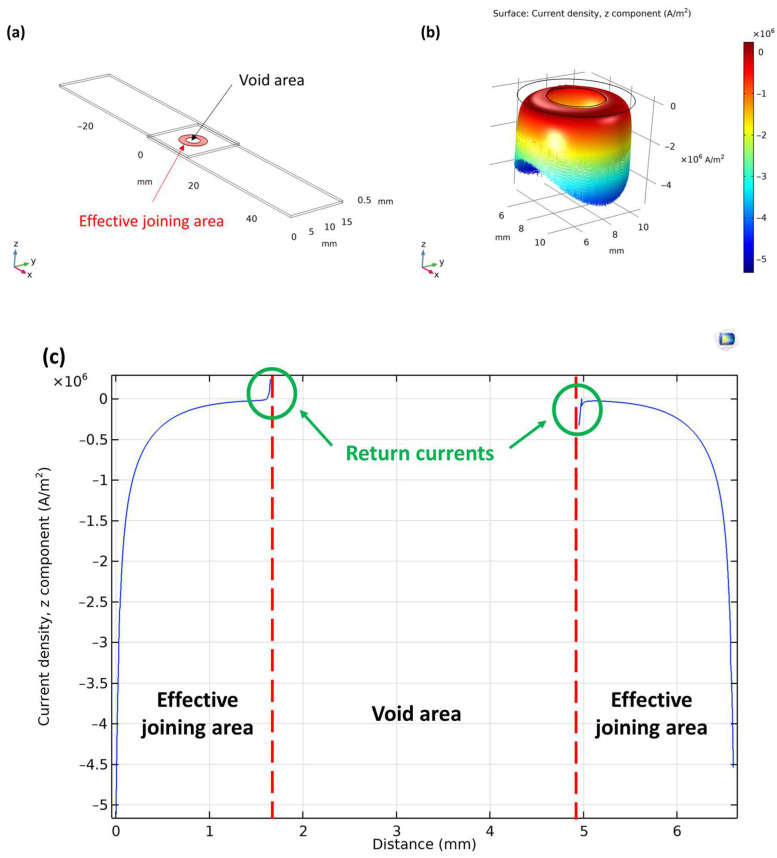
(**a**) The position of the joining area (marked as a red ring) and the void. The appearance of the return current along the circumference of a void in filler material in 2D at the *z* = 0.53 mm section: (**b**); and along the axis at *y* = 7.5 mm: (**c**).

The model calculations give intercepts near zero in *Cases I* and *II* when the presence of voids is not considered (i.e., *A_void_ =* 0). Since, in these cases, both the minima and the maxima are small absolute numbers, the relative changes in *Cases I* and *II* are small as well. The appearance of voids causes a huge change in the intercept of the *R(d)* curve in the near zone. In *Cases III* and *IV*, the high maxima with near zero minima results in the high numbers shown in Figure 12. Simulations of the streamlines within the filler explained the immense effect of the presence of the voids; the reason for the great impact of the voids on the electrical characteristics of the joint is that each void acts as a source of return currents. In the vicinity of voids, the “return current” contributes to the elongation of the current path, thereby leading to an extended interaction of electrons with both the steel sheets and the filler material. Consequently, the resistance experiences an increase, significantly impacting the measured resistance–distance relationship in the near zone. Notably, under conditions of constant void quantity and distribution, the elongation of current paths resulting from these mechanisms remains uniform, thereby rendering it independent of the measurement distance *(d)*. In the context of the *R(d)* function, this phenomenon thus influences the intercept rather than the slope. This effect is exemplified in the vicinity of a single void in Figure 13, where we display the norms of the current density vector (i.e., its magnitude) in the plane midway within the filler layer. In Figure 13c, current density values highlighted in the green circle represent the return current along the circumference of the void indicated by the red vertical dashed line. From the simulated results, it follows that the void area dependence of the intercept in the near zone can be described as

(4) $$i_{n}\approx A_{void}$$

since in *Case V*, both the + and −50% values refer to the presence of voids. The relative change is not so impressive compared to *Cases III* and *IV*; nevertheless, the distribution of the voids has a great influence on the intercept in the near zone.

## 6. Conclusions

This article focuses on the electrical resistance of laser-soldered joints; generally on the challenges associated with the problems of the electrical characterisation, and particularly on the minimisation of the resistive losses in joints. While the electrical characterisation of joints in overlap geometry is pivotal when assessing the performance of a joint, no standardised methods are currently available. The four-point probe method usually employed is ideal for comparative purposes, but it cannot provide exact values for the resistance of the joint.

In order to assess the impact of joint resistance on the overall resistance of the sample, resistance was monitored as a function of the spatial distance between the two inner probes (i.e., those used for voltage measurement). Two zones, the near and far zones, were distinguished depending on whether the voltage probes were placed within or outside the cross-section of the soldered area. Measurements conducted in the far and near zones revealed linear relationships between the resistance and voltage probe distance. A modelling approach using COMSOL Multiphysics provided valuable insight into the complex phenomena and underlying physics. Simulations confirmed the importance of five parameters in determining the electrical resistance of joints; namely, the thickness of the filler material, the effective and the total area, and the area and the position of the voids within the total joint area. The novel approach introduced here revealed how the geometry and the morphology of the bond formed by resolidified filler material influenced the electrical characteristics of a joint. Equation (3) describes the slopes in both the far and near zones, indicating that they are material specific, with the former referring to the material to be joined and the latter including both the sheets and the solder/filler material. The intercepts of the straight lines in the far zone, expressed in ohms, indicate the difference in the resistance of the respective soldered structures as compared to that of the bare metal to be joined. The behaviour of the intercepts also reflects changes in the electrical characteristics of the joints due to inhomogeneities in the number and distribution of the voids.

In summary, this study contributes to the understanding of the effect of the material(s) to be joined and the characteristics of the solder in determining the electrical resistance behaviour in laser-soldered joints. A key, but not the sole beneficiary of these results is the EV sector, where maximising the efficiency and hence minimising the losses of the battery pack could be a game-changer.

## Figures and Tables

**Table 1:** Measured values, and simulation ranges of the five varied properties describing the geometrical characteristics of a laser-soldered sample (laser power of 120 W, irradiation time of 5 s, filler volume of 1.6 mm^3^).

Case	Varied Property	Measured Value	Simulation Range
I	*t_filler_* [mm]	0.047	0.023–0.070
II	*A_eff_* [mm^2^]	34.212	17.106–51.318
III	*A_void_/A_total_*	0.630	0.315–0.945
IV	*A_total_* [mm^2^]	91.562	45.781–137.343
V	*pos_void_* [mm]	6.900	7.500–6.370

**Table 2:** The slope of the *R(d)* curves in the far zone for different metal sheets.

Material	Thickness (mm)	Measured Slope (µΩ/mm)	Resistivity (µΩ)	
			Calculated	Literature Value [30,31]
DC01 steel	0.5	20.259	1.62 × 10^−7^	1.60 × 10^−7^
Stainless steel	0.5	22.543	1.80 × 10^−7^	1.00–7.20 × 10^−7^
Aluminium (99%)	1	1.717	2.74 × 10^−7^	2.65 × 10^−7^
Copper alloy (bronze)	1	4.144	6.63 × 10^−7^	6.00–9.00 × 10^−7^

## Data Availability

The data presented in this study are available on request from the corresponding author. The data are not publicly available due to privacy restrictions.
